# Upregulation of miR-215 attenuates propofol-induced apoptosis and oxidative stress in developing neurons by targeting LATS2

**DOI:** 10.1186/s10020-020-00170-6

**Published:** 2020-05-06

**Authors:** Fang Tang, Lili Zhao, Qi Yu, Tianyin Liu, Hongyan Gong, Zhiyi Liu, Qing Li

**Affiliations:** Department of Anesthesiology, The First Affiliated Hospital of Nanchang University, No. 17 Yongwaizheng Street, Nanchang City, 330006 Jiangxi Province China

**Keywords:** miR-215, LATS2, Propofol, Apoptosis, Oxidative stress

## Abstract

**Background:**

Propofol is an intravenous anesthetic agent that commonly induces significant neuroapoptosis. MicroRNAs (miRNAs) have been reported to participate in the regulation of propofol exposure-mediated neurotoxicity. MiR-215, as one of miRNAs, was found to regulate nerve cell survival. However, the mechanism through which miRNAs regulate propofol exposure-mediated neurotoxicity is still unclear.

**Methods:**

Real-time PCR was used to detect miR-215 expression level. Cell viability was measured using MTT assay. Cell apoptosis was examined via flow cytometry analysis. ROS, MDA, LDH and SOD levels were assayed through ELISA kits. Dual luciferase reporter assay identified the interaction between miR-215 and large tumor suppressor 2 (LATS2). Protein level was detected using western blot analysis.

**Results:**

MiR-215 expression was downregulated in propofol-treated rat hippocampal neurons. MiR-215 mimics promoted cell viability and reduced apoptosis in propofol-treated neonatal rat hippocampal neuron. MiR-215 mimics also caused inhibition of oxidative stress as evidenced by suppression of ROS, MDA and LDH levels as well as increase of SOD level. In addition, we found that large tumor suppressor 2 (LATS2) is a target of miR-215 and miR-215 mimics decreased LATS2 level in propofol-treated neonatal rat hippocampal neuron. Further, LATS2 overexpression suppressed the effect of miR-215 on propofol-induced apoptosis and oxidative stress in neonatal rat hippocampal neuron.

**Conclusion:**

Taken together, we demonstrate that miR-215 attenuates propofol-induced apoptosis and oxidative stress in neonatal rat hippocampal neuron by targeting LATS2, suggesting that miR-215 may provide a new candidate for the treatment of propofol exposure-induced neurotoxicity.

## Background

Propofol is an intravenous anesthetic agent commonly used for the induction and maintenance of anesthesia and sedation (Chidambaran et al. 2015). Propofol can lead to significant neuroapoptosis and affect dendrite development and cognitive function, which causes concern about its safety in pediatric anesthesia (Bosnjak et al. 2016). Reported mechanisms of propofol exposure-induced neurotoxicity include calcium dysregulation, mitochondrial fission, abnormal expression of neurotrophic protein and neuroinflammation (Wei 2011; Cui et al. 2011; Unoki and Nakamura 2001). Therefore, it is necessary to explore the biomarker to prevent and improve propofol exposure-induced neurotoxicity.

MicroRNAs (miRNAs), 18–25 nucleotides in length, are endogenous non-coding RNA molecules that regulate biological processes through suppression of target messenger RNA expression (Fabian et al. 2010; Bartel 2009; Shukla et al. 2011; Ji et al. 2019). Increasing evidences have shown that miRNAs are involved in the regulation of propofol exposure-mediated neurotoxicity. For example, Jiang et al. discovered that miR-141-3p is useful for propofol-mediated suppression of neural stem cells neurogenesis (Jiang et al. 2017). Zheng et al. proved that propofol attenuates neuroinflammatory response of microglia in response to LPS via regulating miR-155 (Zheng et al. 2018). Besides, Zhang et al. verified that propofol anesthesia decreases miR-132 level and inhibits the number of dendritic spines in the hippocampus (Zhang et al. 2017). Interestingly, miR-215 is reduced in ischemic stroke, which leads to suppression of nerve cell apoptosis, autophagy, ischemic infarction and improved neurological deficit via down-regulation of nuclear factor-κB activator 1/ interleukin-17 receptor A pathway. These results suggest that miR-215 plays a neuroprotective role in ischemic injury (Sun et al. 2018). However, the role of miR-215 in propofol exposure-induced neurotoxicity is still unclear.

Large tumor suppressor 2 (LATS2) gene maps onto the human chromosome 13q11–12 (Yabuta et al. 2000). Nuclear LATS2 has been found to activate p53, maintaining the proper chromosome number when mitotic apparatus are impaired (Aylon et al. 2006). Previously, LATS2 was found to promote p53-mediated apoptosis (Aylon et al. 2010) and reduce the expression of BCL-2 and BCL-x(L) (Ke et al. 2004). Moreover, Brandt et al. found that LATS2 is involved in the production of peripheral nerve sheath tumors (Brandt et al. 2019). LATS2 kinase activation inhibits Yap protein to suppress proliferation and cell cycle exit in the process of neurogenesis (Zhang et al. 2012). Notably, LATS2 is predicted to be a target of miR-215 using bioinformatic analysis. Thus, these findings suggest that miR-215 may be involved in regulation of propofol exposure-induced neurotoxicity through LATS2.

In the current study, we investigated miR-215 expression in propofol-treated rat hippocampal neurons. The effects of miR-215 on cell viability and apoptosis were then examined. Furthermore, we explored whether miR-215 modulates propofol exposure-induced neurotoxicity via LATS2. Together these results suggest a new target for the treatment of propofol exposure-induced neurotoxicity.

## Methods

### Neonatal rat hippocampal neuron isolation, culture and transfection

The cell isolation procedures in this study were approved by Guide for the Care and Use of Laboratory Animals and in agreement with the Ethics Committee of The first affiliated hospital of Nanchang University. Neonatal rat hippocampal neuron was isolated as previously reported (Bhargava et al. 2010). Briefly, 1 ~ 2 day-old neonatal Sprague-Dawley rats were sacrificed and the whole brains were collected. The hippocampi were isolated from the neonatal brains and hippocampal neurons were harvested via collagenase digestion of hippocampus tissues. Subsequently, the hippocampal neurons were cultured as a monolayer at 37 °C with a normoxic 95% air and 5% CO_2_ incubator. The hippocampal neurons (1 × 10^7^ cells/well) were seeded in 24-well plates and transfected with 50 nM miR-215 mimics (miR-215) or miR-215 negative control (miR-NC) after 3 days using Lipofectamine 2000 reagent. After transfection for 48 h, the cells were treated with 20 μM propofol for 0, 2, 4, 6 or 12 h under a normoxic 95% air and 5% CO_2_ incubator. Finally, the cells were examined in the following experiments.

### Real-time PCR

Total RNA was isolated via TRIzol reagent (Invitrogen,Carlsbad,CA,USA). Synthesis of cDNA was conducted using TaqMan miRNA reverse transcription kit (Beyotime, Shanghai, China). The real-time PCR was carried out using SYBR Green (Takara, Dalian, China) on an ABI 7300 instrument. The miR-215 expression level was calculated via the 2^−ΔΔCt^ method. The U6 was used as internal control. The primers used for this study were as follows: miR-215-F: AUGACCUAUGAAUUGACAGAC, miR-215-R: UCUGUCAUUUAGGCCAAUA. U6-F: GCTTCGCAGCACATATACTAAAAT, U6-R: CGCTTCACGAATTTGCGTGTCAT.

### MTT assay

A total of 3000 cells in 200 μl of medium were plated into 96-well plates. The cells were incubated with 0.5 mg/ml 3-(4,5-dimethyl-2-thiazolyl)-2,5-diphenyl-2-H-tetrazolium bromide (MTT) solution (Beyotime, Shanghai, China) in 200 μl of medium for 4 h. The absorbance value was measured at a wavelength of 490 nm.

### Apoptosis assay

Cells (10^5^ cells) were collected and suspended in Annexin V incubation solution. Then, the cells were stained with 5 μl Annexin V-fluorescein isothiocyanate (FITC) and 5 μl propidium iodide (PI) solution (Beyotime, Shanghai, China) in the dark for 20 min. Apoptosis was then analyzed using flow cytometry.

### Oxidative stress and ROS measurement

Transfected cells were treated with 20 μM propofol for 6 h under a normoxic 95% air and 5% CO_2_ incubator and then collected. Subsequently, the malondialdehyde (MDA), lactate dehydrogenase (LDH) and superoxide dismutase (SOD) were detected via ELISA kits (NJJC Bio Engineering Institute, Nanjing, China). The reactive oxygen species (ROS) level was examined in cells using 2′,7′-dichlorofluorescin diacetate (DCFDA) for 30 min at 37 °C. The cells were observed, and data were analyzed through microplate reader.

### Dual luciferase reporter assay

The wide-type 3′-UTR sequence of LATS2 contained the miR-215 binding site. Then, site-directed mutagenesis of the putative target site for miR-215 in wide-type 3′-UTR sequence of LATS2 was performed to generate the mutant-type 3′-UTR sequence and the site-directed mutagenesis was showed in Fig. 3a. The primers designed by primer premier 5.0 was used to conduct amplification. The thermal cycle profile was as follows: denaturation for 20 s at 95 °C, annealing for 30 s at 54 °C, and extension for 40 s at 72 °C. Subsequently, the sequences were inserted into the pmirGLO reporter vector (Promega, Shanghai, China) between *XhoI* and *SalI* restriction enzyme sites using T4 DNA Ligase to generate luciferase reporter constructs, and named by LATS2-WT and LATS2-MUT. Nucleotide sequences of the constructs were identified through DNA sequencing. HEK293T cells were co-transfected with 50 ng of LATS2-WT or LATS2-MUT and 20 μM of miR-215 or miR-NC for 48 h. Finally, the luciferase activity was determined via dual luciferase reporter assay system (Promega, Shanghai, China).

### Western blot analysis

Proteins were extracted via RIPA lysis buffer (Beyotime, Shanghai, China). The concentration was quantified by a BCA kit (Beyotime, Shanghai, China). Proteins (45 μg) were separated by SDS-PAGE and transferred to polyvinylidene difluoride membrane (Millipore, USA). After blocking with 5% non-fat milk for 1 h at 37 °C, the membrane was incubated with antibodies against LATS2 (1:1000, Abcam, Shanghai, China) and β-actin (1:1000, Abcam, Shanghai, China) overnight at 4 °C. Subsequently, the secondary antibody (1:3000, Beyotime, Shanghai, China) was used to incubate the membrane for 2 h at 37 °C. The signals were visualized via an enhanced chemoluminescence kit (Takara, Dalian, China). The blots were analyzed with ImageJ software.

### Statistical analysis

The data were presented as means ± SD and analyzed with SPSS 18.0. Statistical analysis was conducted via two-tailed unpaired Student’s t-test or one-way ANOVA with Tukey’s test. *P* value less than 0.05 was considered as statistically significant difference.

## Results

### Effect of miR-215 on propofol-induced apoptosis in neonatal rat hippocampal neuron

**Fig. 1:**
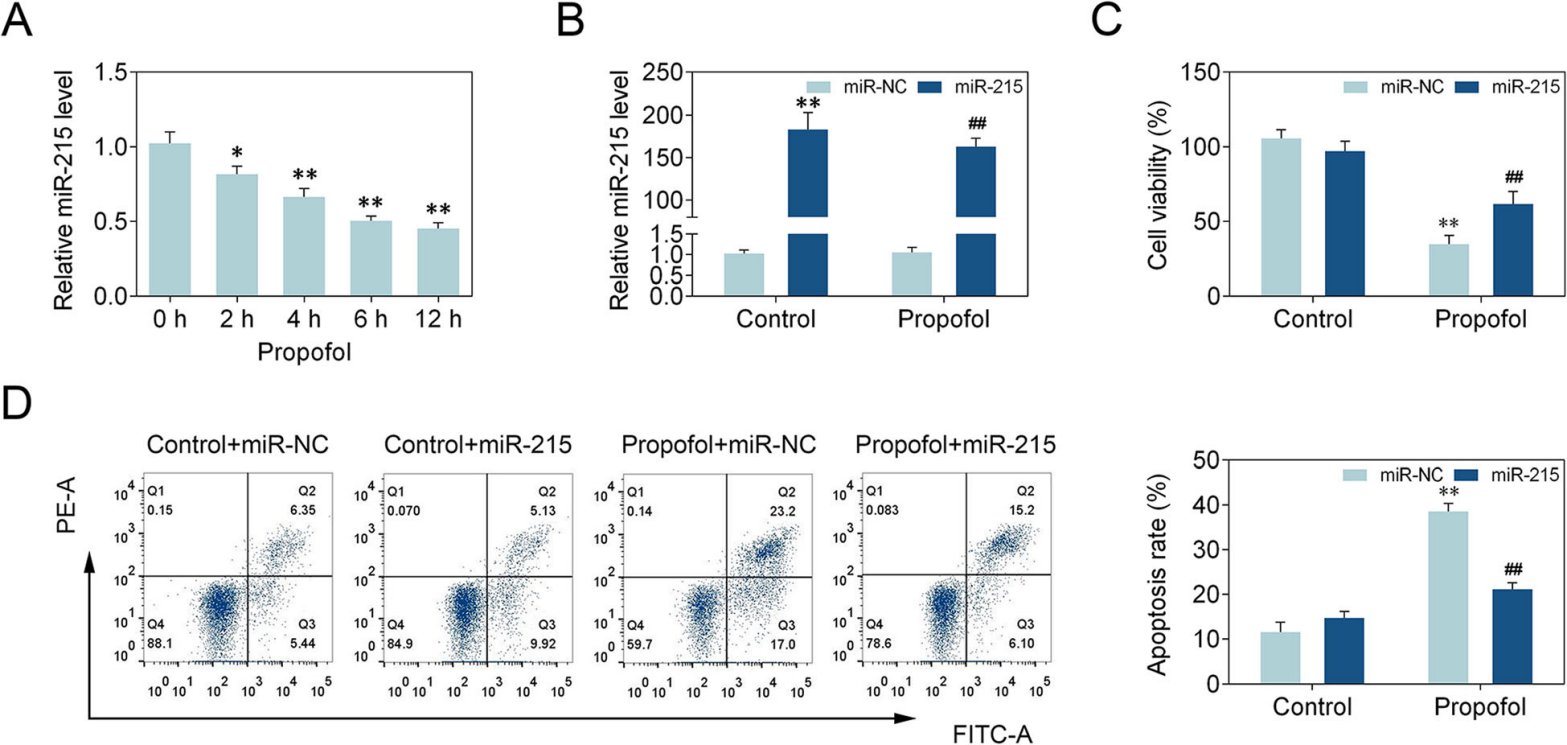
Effect of miR-215 on propofol-induced apoptosis in neonatal rat hippocampal neuron. **a** miR-215 level was measured using real-time PCR. *n* = 3. *, *p* < 0.05. **, *p* < 0.01. * vs 0 h. Statistical analysis was conducted via one-way ANOVA by Tukey’s test. **b** miR-215 level was detected via real-time PCR. Statistical analysis was conducted via one-way ANOVA by Tukey’s test. **c** MTT assay was used to determine cell viability. Statistical analysis was conducted via one-way ANOVA by Tukey’s test. **d** Flow cytometry analysis was used to detect apoptosis. Statistical analysis was conducted via one-way ANOVA by Tukey’s test. *n* = 3. **, *p* < 0.01. ##, *p* < 0.01. * vs Control+NC mimics. # vs Propofol+NC mimics

To explore the effect of miR-215 on propofol-induced apoptosis, we first examined miR-215 expression in neonatal rat hippocampal neuron. Real-time PCR showed that propofol treatment decreased miR-215 level in a time-dependent manner (Fig. 1a). MiR-215 level was increased in miR-215 mimics transfected neonatal rat hippocampal neuron treated with propofol (Fig. 1b). MTT assay demonstrated that propofol treatment reduced cell viability, whereas miR-215 mimics enhanced cell viability (Fig. 1c). In addition, apoptosis was increased by propofol treatment and miR-215 mimics suppressed propofol-induced apoptosis (Fig. 1d). These results indicate that miR-215 has a suppressive role in propofol-induced apoptosis in neonatal rat hippocampal neuron.

### Effect of miR-215 on propofol-induced oxidative stress in neonatal rat hippocampal neuron

**Fig. 2:**
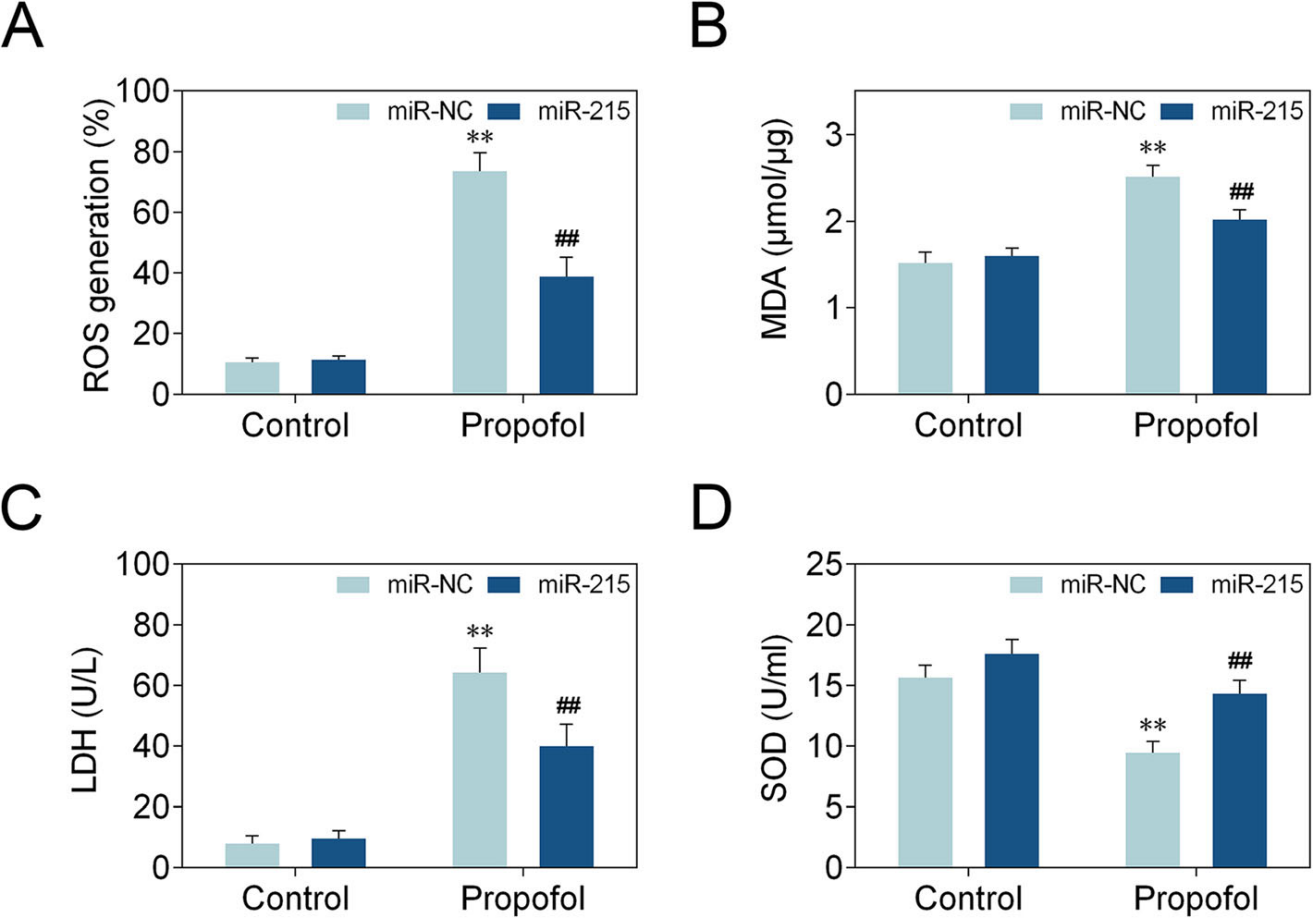
Effect of miR-215 on propofol-induced oxidative stress in neonatal rat hippocampal neuron. **a** ROS level was examined in neonatal rat hippocampal neuron. Statistical analysis was conducted via one-way ANOVA by Tukey’s test. **b** MDA detection was conducted in neonatal rat hippocampal neuron. Statistical analysis was conducted via one-way ANOVA by Tukey’s test. **c** LDH assay examined the LDH level in neonatal rat hippocampal neuron. Statistical analysis was conducted via one-way ANOVA by Tukey’s test. **d** SOD level from neonatal rat hippocampal neuron was assayed. Statistical analysis was conducted via one-way ANOVA by Tukey’s test. *n* = 3. **, *p* < 0.01. ##, *p* < 0.01. * vs Control+NC mimics. # vs Propofol+NC mimics

We then examined the role of miR-215 in propofol-induced oxidative stress. We first analyzed ROS level and the results showed that ROS generation was suppressed by miR-215 mimics in propofol-treated neonatal rat hippocampal neuron (Fig. 2a). MDA and LDH assays revealed that miR-215 mimics decreased MDA and LDH levels (Fig. 2b, c). On the other hand, miR-215 mimics increased SOD level (Fig. 2d). These findings suggest that miR-215 reduces propofol-induced oxidative stress in neonatal rat hippocampal neuron.

### LATS2 is a target of miR-215

**Fig. 3:**
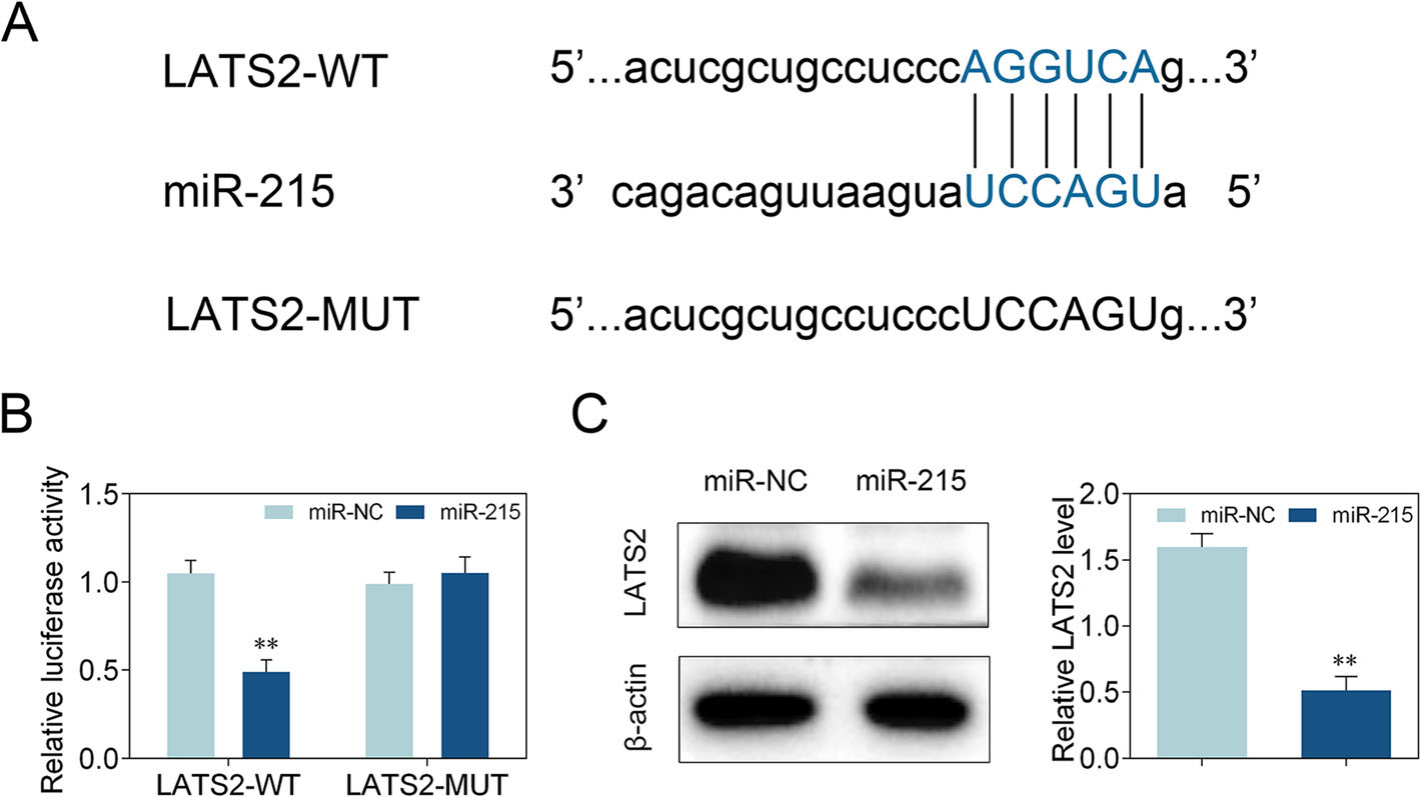
LATS2 is a target of miR-215. **a** TargetScan analysis was used to predict the binding site of miR-215. **b** Dual luciferase reporter assay confirmed the interaction between miR-215 and LATS2. Statistical analysis was conducted via one-way ANOVA by Tukey’s test. **c** Western blot analysis detected LATS2 protein level in neonatal rat hippocampal neuron. Statistical analysis was carried out through two-tailed unpaired Student’s t-test. *n* = 3. **, *p* < 0.01

TargetScan analysis (http://www.targetscan.org/vert_72/) was used to predict the binding site of miR-215. The results showed that LATS2 is a target of miR-215 (Fig. 3a). To confirm the interaction between miR-215 and LATS2, dual luciferase reporter assay was performed and showed that miR-215 mimics decreased the relative luciferase activity of LATS2-WT. However, there was no effect on the relative luciferase activity in HEK293T cells co-transfected with miR-215 mimics and LATS2-MUT (Fig. 3b). Western blot analysis verified that miR-215 mimics inhibited LATS2 protein level in neonatal rat hippocampal neuron (Fig. 3c). The data indicated that LATS2 is a target of miR-215 and miR-215 represses LATS2 level.

### Downregulation of LATS2 induced by miR-215 overexpression in propofol-treated neonatal rat hippocampal neuron

**Fig. 4:**
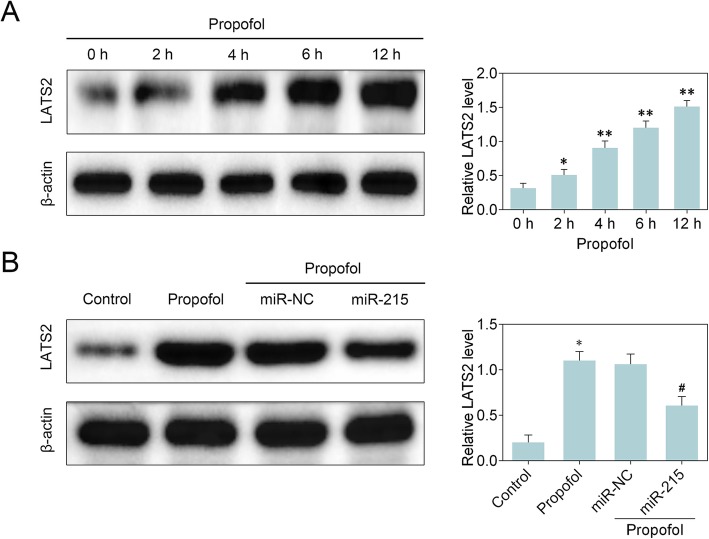
Downregulation of LATS2 induced by miR-215 in propofol-treated neonatal rat hippocampal neuron. **a** LATS2 protein level was measured via western blot analysis in neonatal rat hippocampal neuron. Statistical analysis was conducted via one-way ANOVA by Tukey’s test. *n* = 3. *, *p* < 0.05. **, *p* < 0.01. * vs 0 h. **b** Western blot analysis detected LATS2 protein level in neonatal rat hippocampal neuron. Statistical analysis was conducted via one-way ANOVA by Tukey’s test. *n* = 3. *, *p* < 0.05. #, *p* < 0.05. * vs Control. # vs propofol+NC mimics

We then examined the effect of miR-215 overexpression on LATS2 in propofol-treated neonatal rat hippocampal neuron. Western blot analysis demonstrated that LATS2 protein level was elevated in neonatal rat hippocampal neuron with propofol treatment in a time-dependent manner (Fig. 4a). MiR-215 overexpression exhibited a suppressive role in propofol-induced increase of LATS2 level using western blot analysis (Fig. 4b). These results indicate that miR-215 overexpression downregulated LATS2 level in propofol-treated neonatal rat hippocampal neuron.

### MiR-215 attenuates propofol-induced apoptosis and oxidative stress in neonatal rat hippocampal neuron by targeting LATS2

**Fig. 5:**
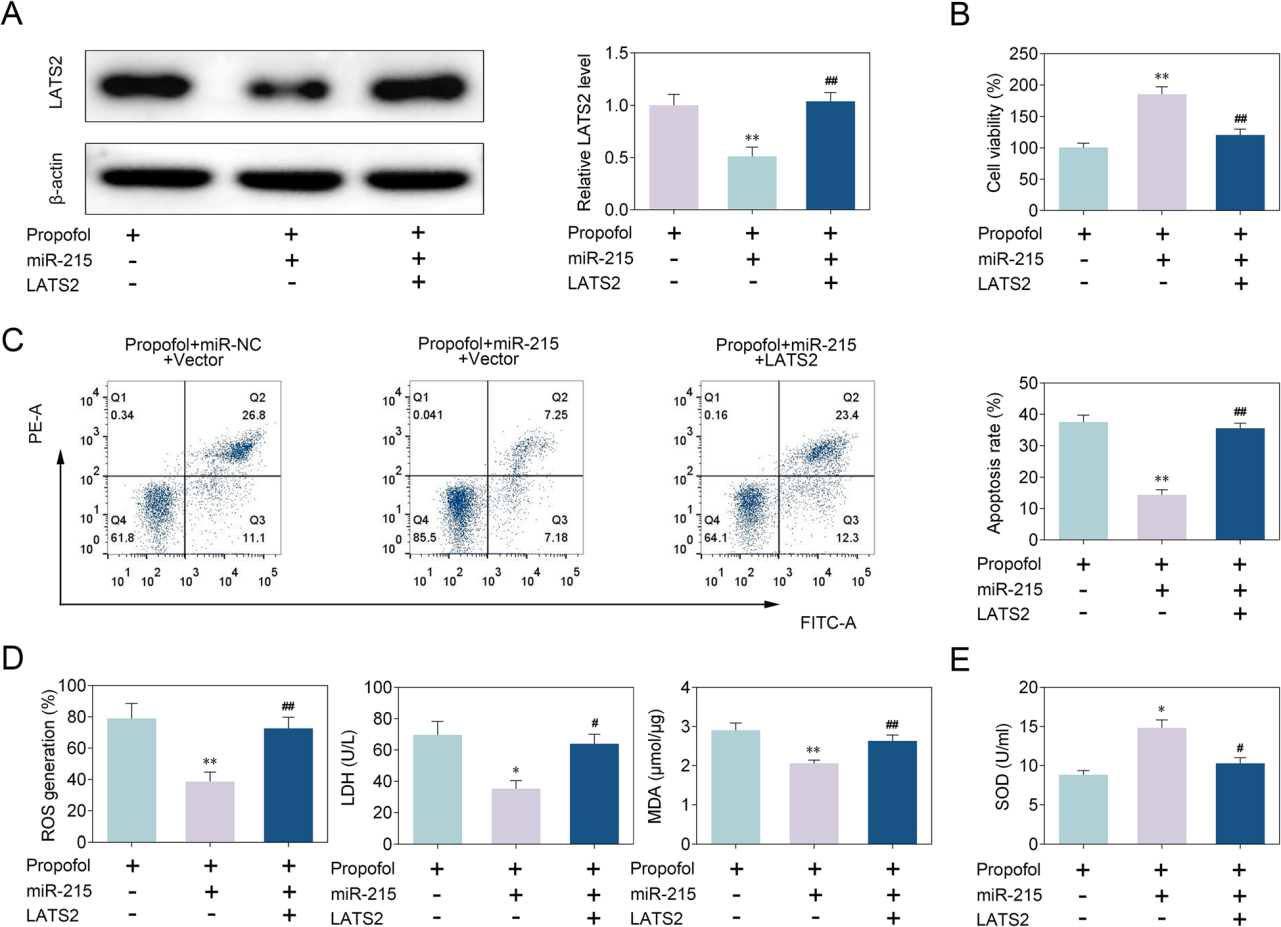
MiR-215 attenuated propofol-induced apoptosis and oxidative stress in neonatal rat hippocampal neuron by targeting LATS2. **a** Western blot analysis revealed LATS2 protein level. Statistical analysis was conducted via one-way ANOVA by Tukey’s test. **b** Cell viability was examined using MTT assay. Statistical analysis was conducted via one-way ANOVA by Tukey’s test. **c** Flow cytometry analysis was used to detect apoptosis. Statistical analysis was conducted via one-way ANOVA by Tukey’s test. **d** Levels of ROS, LDH, MDA and SOD were analyzed in neonatal rat hippocampal neuron. Statistical analysis was conducted via one-way ANOVA by Tukey’s test. *n* = 3. *, *p* < 0.05. **, *p* < 0.01. #, *p* < 0.05. ##, *p* < 0.01. * vs Propofol+miR-NC + Vector. # vs Propofol+miR-215 + Vector

To test the hypothesis that miR-215 affects propofol-induced apoptosis and oxidative stress by targeting LATS2, we co-transfected miR-215 mimics and LATS2 in neonatal rat hippocampal neuron. Western blot analysis revealed that miR-215 mimics inhibited LATS2 protein level, whereas LATS2 overexpression elevated the protein level (Fig. 5a). Moreover, LATS2 overexpression suppressed the increase of cell viability induced by miR-215 mimics (Fig. 5b). Flow cytometry analysis showed that miR-215 mimics inhibited apoptosis, whereas LATS2 overexpression abrogated miR-215 induced inhibition of apoptosis (Fig. 5c). Furthermore, miR-215 mimics suppressed ROS, MDA and LDH levels, and promoted SOD level. On the other hand, LATS2 overexpression increased ROS, MDA and LDH levels, and decreased SOD level (Fig. 5d, e). These data suggest that miR-215 attenuates propofol-induced apoptosis and oxidative stress in neonatal rat hippocampal neuron by targeting LATS2.

## Discussion

In this study, we found that miR-215 was decreased and LATS2 was increased in propofol-treated neonatal rat hippocampal neuron. Functional analysis showed that miR-215 has a suppressive role in propofol-induced apoptosis and oxidative stress in neonatal rat hippocampal neuron. Furthermore, we demonstrated that LATS2 is a target of miR-215, and miR-215 could reduce propofol-induced LATS2 level. LATS2 overexpression suppressed the effect of miR-215 on propofol-induced apoptosis and oxidative stress in neonatal rat hippocampal neuron. These data imply that miR-215 participates in the regulation of propofol-induced apoptosis and oxidative stress in neonatal rat hippocampal neuron by targeting LATS2.

Previous studies have shown that miRNAs are implicated in neurological diseases (Johnson et al. 2008; Asikainen et al. 2010; Hebert et al. 2008). Moreover, miRNAs dysregulation plays an important role in neurotoxicity (Kaur et al. 2012). Recently, miR-665 was found to be significantly increased in primary cultured astrocytes treated with propofol, and it inhibited BCL2L1 (Bcl-xl) (a suppressor of apoptosis) (Sun and Pei 2016). Moreover, miR-34a was discovered to be elevated after propofol treatment, and miR-34a knockdown could inhibit propofol-induced apoptosis (Li et al. 2018). Some miRNAs were reported to reverse the propofol-mediated effect. Wang et al. proved that miR-383 was downregulated by propofol treatment, and it could alter the propofol-induced upregulation of hippocampal neuron apoptosis (Wang et al. 2018a). Twaroski et al. found that miR-21 was decreased in the neurons, and miR-21 overexpression alleviated propofol-caused cell death in human embryonic stem cells-derived neurons (Twaroski et al. 2014). Consistent with the later studies, our study revealed that miR-215 was downregulated in propofol-treated neonatal rat hippocampal neuron, and miR-215 mimics inhibited propofol-induced apoptosis. These results imply that miR-215 may act as a suppressive factor in propofol-induced apoptosis in neonatal rat hippocampal neuron.

Oxidative and antioxidative balance is disrupted upon oxidative stress. Oxidative and antioxidative products produced in cells include MDA, LDH, and SOD (Rodrigo et al. 2016). It is reported that the balance between ROS production and scavenging is important in oxidative stress and has protective or damaging effects in several diseases (Aon et al. 2010). Oxidative stress and ROS have been found to be associated with a number of physiological and pathological processes (Huang et al. 2009). Increasing evidences have revealed that a large number of ROS could directly or indirectly induce oxidative damage to cells (Wang et al. 2018b; Lee et al. 2015). Notably, in our study, we discovered that miR-215 mimics reduced ROS, MDA and LDH levels, and increased SOD generation in propofol-treated neonatal rat hippocampal neuron, suggesting that miR-215 can negatively regulate propofol-induced oxidative stress in neonatal rat hippocampal neuron.

Accumulating evidences have shown that miRNAs carry out their functions by targeting mRNAs (Bartel 2009). Previous study proved that miR-410-3p has a neuroprotective effect on sevoflurane anesthesia-induced cognitive dysfunction by targeting C-X-C motif chemokine receptor 5 (Su et al. 2019). In addition, miR-133a-5p is involved in the protective effect of propofol-mediated hepatic ischemia/reperfusion injury through targeting MAPK6 (Hao et al. 2017). MiR-665 was reported to participate in the neurotoxicity induced by propofol via targeting Bcl-2-like protein 1 BCL2L1 (Sun et al. 2015). Here we demonstrate that miR-215 could target LATS2. Moreover, the propofol-treated neonatal rat hippocampal neuron co-transfected with miR-215 mimics and LATS2 overexpression led to the suppression of miR-215 induced increase of cell viability and decrease of apoptosis and oxidative stress. The data indicate that miR-215 can attenuate propofol-induced apoptosis and oxidative stress in neonatal rat hippocampal neuron by targeting LATS2. However, to better clarify the role of miR-215 in neonatal rat hippocampal neuron by targeting LATS2, the function of miR-215 inhibition and LATS2 silencing in chemical-induced neonatal rat hippocampal neuron with high miR-215 expression will be performed in the near future.

## Conclusion

In conclusion, in the current study we found that downregulation of miR-215 and upregulation of LATS2 were induced by propofol. Additionally, miR-215 overexpression alleviated propofol-induced apoptosis and oxidative stress in neonatal rat hippocampal neuron by targeting LATS2. Our results suggest miR-215 may provide a new therapeutic target to treat propofol-induced neuroapoptosis in developing neurons.

## Data Availability

All data generated or analyzed during this study are included in this published article.

## Abbreviations

miRNAs: MicroRNAs
LATS2: large tumor suppressor 2
